# Preparation of a bionic lotus leaf microstructured surface and its drag reduction performance

**DOI:** 10.1039/d2ra01495e

**Published:** 2022-06-06

**Authors:** Huan Wang, Guihang Luo, Lei Chen, Yuqiu Song, Cuihong Liu, Liyan Wu

**Affiliations:** College of Engineering, Shenyang Agricultural University Shenyang 110866 P. R. China wly78528@syau.edu.cn; Key Laboratory of Bionic Engineering, Ministry of Education, Jilin University Changchun 130022 P. R. China

## Abstract

Reducing machinery surface friction resistance can improve the efficiency of energy utilization. The lotus leaf, as everyone knows, has a strong capacity for self-cleaning and hydrophobicity. In this paper, the bionic surface of the lotus leaf was prepared in large-area, and its drag reduction performance was studied by both numerical simulation and experimental analysis. Experimental results showed that the maximum drag reduction rate of the bionic surface was 6.29% which appeared at a velocity of 3 m s^−1^. The contact state between liquid and bionic surface changed from Cassie state to Wenzel state with the increase of water flow velocity. The surface free energies of the bionic surface and smooth surface were 1.09 mJ m^−2^ and 14.26 mJ m^−2^, respectively. In the droplet rolling experiment, the water droplet was a hemisphere when it rolled on the smooth surface, while it was an ellipsoid on the bionic surface. This study provides a theoretical basis for the structural design of bionic drag reduction surfaces, which are expected to be applied in underwater vehicles.

## Abstract 2

The bionic surface of the lotus leaf was prepared in large-area, and its drag reduction performance was studied by both numerical simulation and experimental analysis.

## Graphical abstract

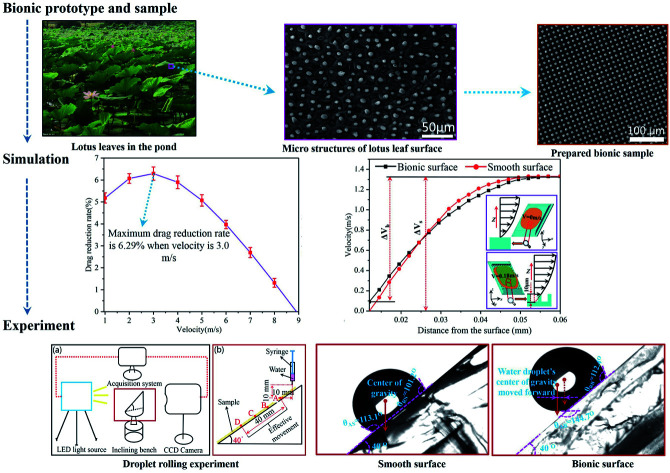

## 1. Introduction

It is well known that the self-cleaning capacity and hydrophobicity of lotus leaves are related to the wax coat and papillae structure. The micro/nano hierarchy morphology of papillae can impact the surface energy of lotus leaves.^1,2^ In previous reports, the interface with low surface energy has superior performance in antifouling,^3^ anti-icing,^4^ antifogging,^5^ antireflection,^6^ oil-water separation,^7^ and so on. These extraordinary properties have stimulated the interest of researchers. How to prepare a superior performance bionic lotus leaf surface has been a research topic for years.^8^ By the chemical vapor deposition method, Wang *et al.* prepared a lotus-leaf-like superhydrophobic carbon nanotube film with micro/nanoscale hierarchical structure, which could effectively prevent the small water droplets from penetrating the film.^9^ By the nanosecond laser processing method, Hauschwitz *et al.* prepared lotus leaf double-scale structures on an aluminium alloy.^10^ By the multiscale stereolithography method, Li *et al.* prepared artificial lotus leaves with micropillar structure.^11^ Das *et al.* prepared microstructures of lotus leaves by direct replication. However, the disadvantage of this method was that it was difficult to demould, and the size of the sample was limited by the size of the lotus leaf.^12^ Bhushan *et al.* prepared a lotus-leaf-like surface by replicating micro patterns on silicon surfaces, and the nanostructures were created by self-assembly of wax platelets (*n*-hexadecane) and tubules (*T. majus* and lotus).^13^ Rong *et al.* prepared micro/nanostructures of lotus leaves on metal substrates by using a laser ablation method.^14^

Reducing energy consumption has attracted widespread attention. The drag reduction function of the superhydrophobic surface has increasingly attracted the interest of scholars and become a popular topic. The lotus-leaf-like superhydrophobic surface showed good drag reduction performance in previous studies.^15,16^ Rong *et al.* analyzed the drag reduction performance of bionic lotus leaf surface by both numerical simulation and experiment analysis, and the maximum drag reduction rate reached 52.76% at velocity of 1.55 m s^−1^.^14^ Sheng *et al.* studied the sustainability of the air layer on the lotus leaf surface and believed that the superhydrophobic surface can maintain a stable layer of air.^17^ Lyu *et al.* showed that drag reduction of the superhydrophobic surface was mainly caused by the velocity slip of the fluid on solid–liquid interfaces.^18^ Lu *et al.* explored the drag reduction mechanism of the bionic lotus leaf surface through the volume of the fluid simulation way, and the microstructure size with the optimal lubrication was obtained.^19^

From all the studies above, several relevant issues remain unresolved. For example, the contact states between water flow and hydrophobic surface at different speeds have not been explored. The area of the prepared bionic surface is too small to be applied in engineering, and the processing techniques are complicated. In this work, the drag reduction mechanism of the hydrophobic surface was studied by combining numerical simulation with actual experiment. The contact states between water flow and the hydrophobic surface under different speeds were explored, and the relationship between drag reduction rate and water flow velocity was established by numerical analysis. The bionic surface was prepared by imitating the lotus leaf surface microstructure. The wettability and drag reduction performance of the bionic surface were analyzed, and the shape of the rolling water droplet was observed through the inclining bench experiment. The prepared bionic surface is expected to be utilized for the surface of underwater vehicle. This study provides a theoretical basis for the drag reduction research of hydrophobic surface.

## 2. Experiments

### 2.1 Materials

Lotus leaf was collected from Nanhu Lake, Changchun, China. Polydimethylsiloxane (PDMS) was obtained from Wuxi Fittes International Trade Limited Co., Ltd. PDMS is a kind of non-toxic, anti-wear, hydrophobic, and low surface energy polymer materials, which is widely used in cosmetics, brighteners, lubricants, release agents, water proof agent, *etc.*^20–22^ The silicon wafer and mask plate were purchased from Suzhou Institute of Nano-Tech and Nano-Bionics, Chinese Academy of Sciences.

### 2.2 Microstructure of lotus leaves surface

**Fig. 1:**
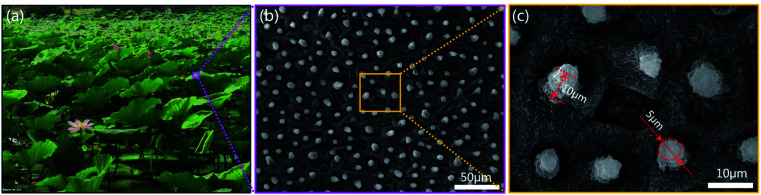
The structure of lotus leaf. (a) Lotus leaves in the pond. (b) SEM image of the lotus leaf. (c) Magnified papilla microstructures.

Fig. 1(a) shows lotus leaves in the pond. The microstructure of lotus leaf surface was observed by scanning electron microscope (SEM), as shown in Fig. 1(b) and (c). There are many papilla microstructures on the surface of lotus leaf, as shown in Fig. 1(b), and the papilla structure resembles cylinder. Fig. 1(c) shows the magnified papilla structures. The diameter of papillae ranges from 2 μm to 8 μm, and the height ranges from 6 μm to 12 μm.

### 2.3 Establish of the simulation model

**Fig. 2:**
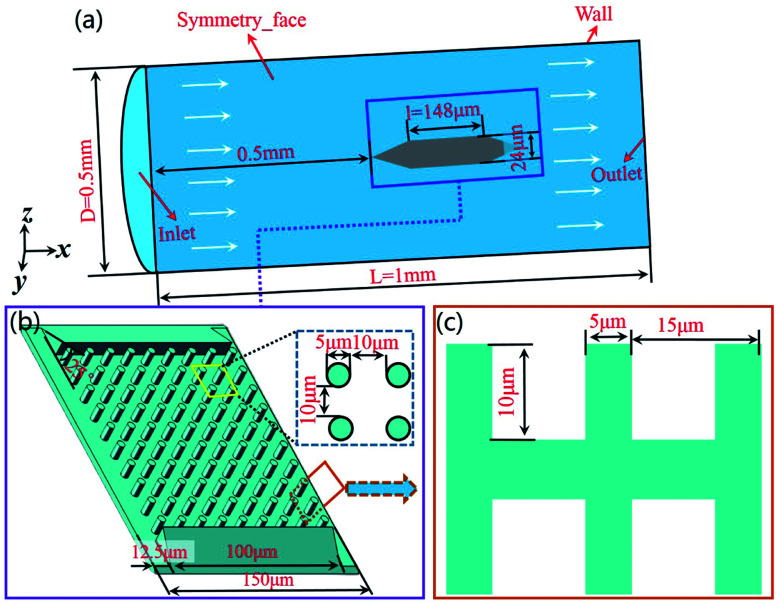
Establishment of simulation model. (a) The flow field model for numerical simulation. (b) The sample structure for numerical simulation. (c) The size of microstructure.

The simulation model was established for analysis of resistance and drag reduction mechanism, as shown in Fig. 2(a). In order to make it easier to build the 3D models and improve computing speed, the papillae structure on the surface of lotus leaf is regarded as cylinders structure in the numerical simulation, as shown in Fig. 2(b) and (c). The diameter of the cylinder is 5 μm, the spacing between two adjacent cylinders is 10 μm, and the height is 10 μm. The diameter of the flow field is 0.5 mm and the length is 1 mm. The height of the sample is 24 μm, the length is 148 μm, and the width is 150 μm.

In order to avoid the influence of the inlet effect on the flow field (Fig. 2(a)), the distance between the sample and the inlet is set as 0.5 mm. The fluid medium is water. The density is 998.2 kg m^−3^, and the dynamic viscosity coefficient is 1.0 × 10^−3^ Pa s. The hydraulic diameter of the model is 0.5 mm, and the wall is set as a non-slip wall. Turbulent dissipation will occur when the fluid flows through the sample surface. Therefore, the *k*–*ε* equation was selected in this simulation.^23^ The turbulent kinetic energy and turbulent intensity of the sample were calculated according to eqn (1)–(3).

1 *I* = 0.16(Re)^−1/8^ (Re = *ρuL*/*μ*)

2
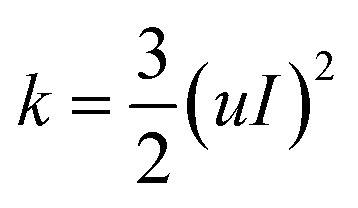
3
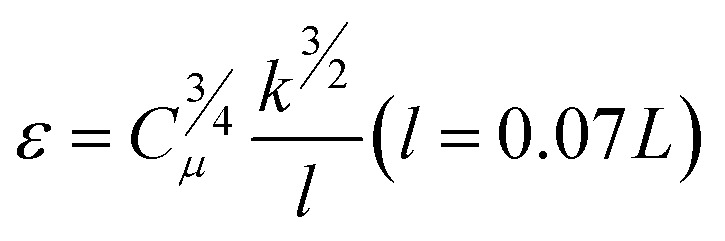
where *I* represents the turbulence intensity. Re is the Reynolds number, *ρ* the fluid density. *u* is the mean velocity of fluid. *k* is the turbulence kinetic energy and *μ* is the viscosity coefficient. *L* is the characteristic length. *C*_*μ*_ = 0.09.

### 2.4 Fabrication of imitating lotus leaf surface

**Fig. 3:**
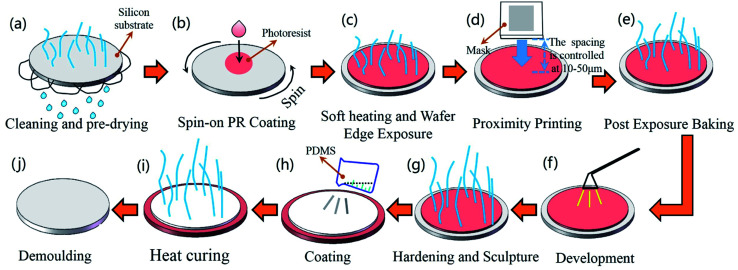
Fabrication process of bionic surface.

The bionic samples were fabricated by the combination method of photolithography and vacuum casting. The sample preparation steps are as follows (Fig. 3).

(a) Cleaning and preheating: to clean contaminants on the surface of the silicon substrate, the substrate was washed in deionized water for 1 to 2 minutes. Then, it was heated in a vacuum drying oven for 2 to 3 minutes.

(b) Spin-on PR coating: dropping the photoresist on the silicon substrate. The rotation speed was gradually increased from 500 rpm to 3000 rpm.

(c) Soft heating and wafer edge exposure: the silicon substrate coated with photoresist was heated in a vacuum drying oven for 30–60 seconds at 95 °C, and the pressure was controlled between 0.1–0.3 MPa.

(d) Proximity printing: the distance between mask and photoresist was maintained in the range of 10–50 μm.

(e) Post exposure heating: adjusting the temperature of the vacuum drying oven to 110–130 °C and keep it for 5 minutes.

(f) Development: the silicon substrate was coated again with (tetramethylammonium hydroxide) on the layer of photoresist.

(g) Hardening and sculpture: to completely remove the solvent in the photoresist and obtain the negative template of papilla microstructure.

(h) Coating: spraying a layer of release agent on the negative template. Mixing Sylguard-184A and Sylguard-184B in a ratio of 10 to 1. Then, dropping 20 mL of the mixture on the surface of negative template.

(i) Heat curing: placing the negative template in a vacuum drying oven for 1 hour. The pressure was maintained at 0.2–0.4 MPa and the temperature was maintained at 70 °C.

(j) Finally, the template was demoulded, the bionic surface with papillae microstructure was obtained.

### 2.5 Experiment of the inclining bench

**Fig. 4:**
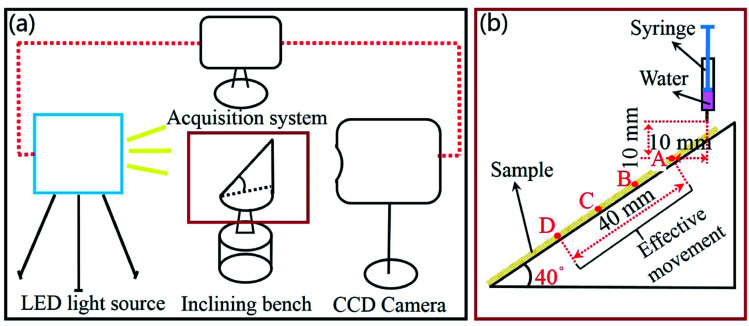
Droplet rolling experiment. (a) Schematic diagram of the experimental device. (b) Inclining bench.

When a droplet moves on a inclining bench, the movement of the center of gravity of the droplet can be used to analyze the effect of drag reduction.^24^ In this study, the inclining bench experiment was carried out at 25 °C, and the angle of the inclining bench was 40°, as shown in Fig. 4(a). The smooth surface and bionic surface were pasted onto the inclining bench, as shown in Fig. 4(b). The water droplet volume is 0.01 mL. Both the horizontal and vertical distances of the syringe from point A are 10 mm.

### 2.6 The tunnel experiment

**Fig. 5:**
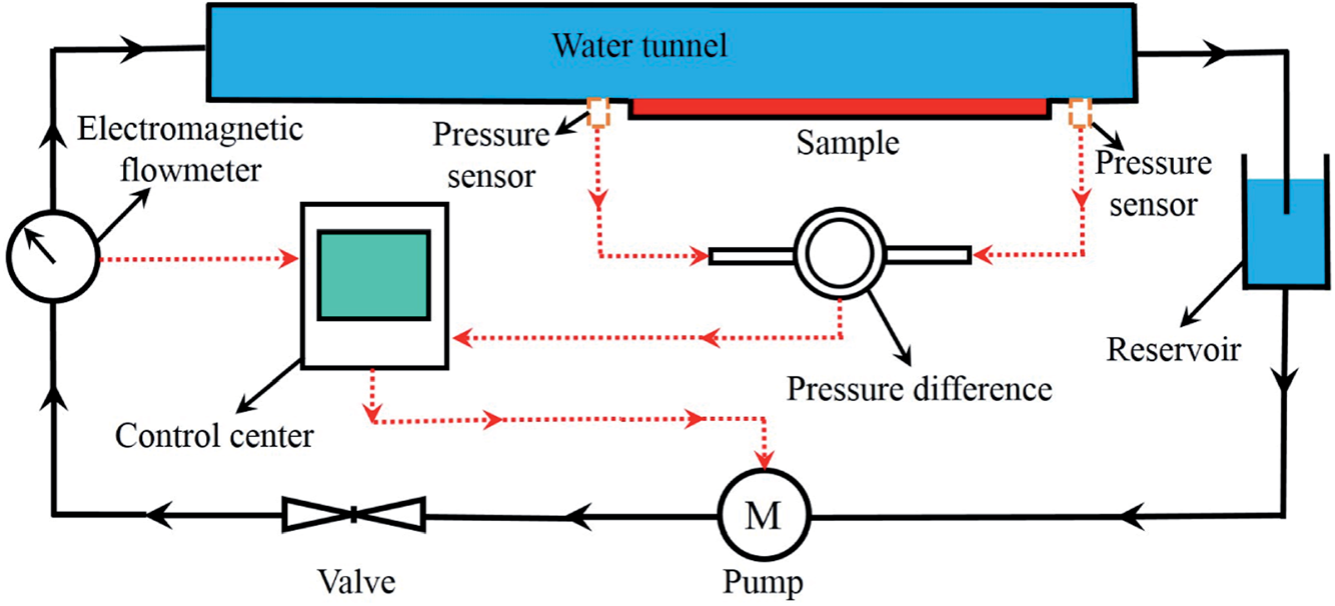
Schematic diagram of the water tunnel experiment device.

The drag reduction effect of the bionic surface was tested through the water tunnel experiment. Fig. 5 shows the schematic diagram of the experimental device. The velocity of water flowing was controlled by the valve, and the resistance of the sample was calculated by comparing the pressure before and after the sample.

## 3. Results and discussion

### 3.1 Properties of the bionic sample

**Fig. 6:**
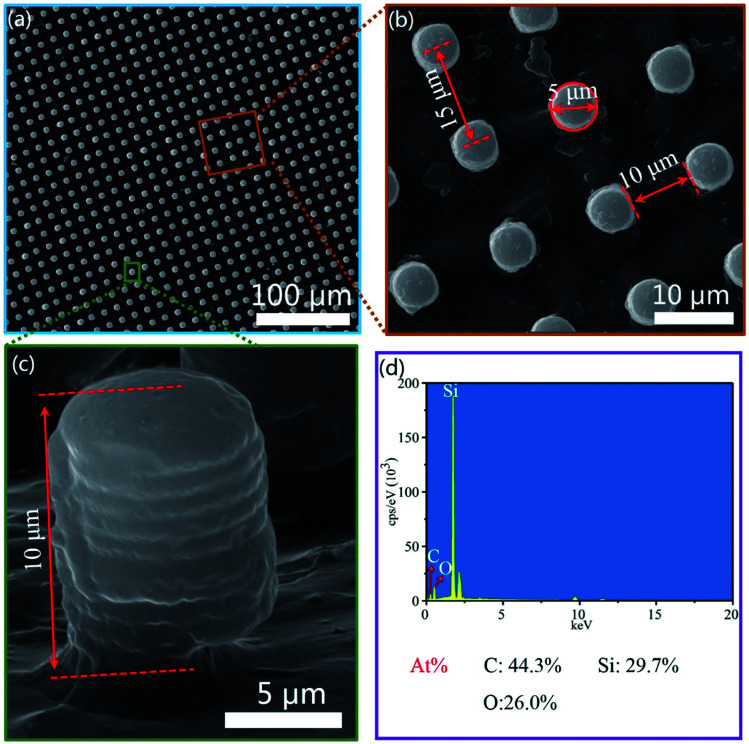
The morphologies and elements distribution on the bionic sample surface. (a and b) The micro morphologies of the bionic surface at 1000 and 5000 times magnification. (c) The cross section structure of the bionic surface. (d) The elements distribution of ESD results.

The size of the bionic sample is 50 mm × 50 mm. The morphology and elements of the bionic surface were analyzed by SEM and Energy Dispersive Spectroscopy (EDS). From the Fig. 6(a) and (b), the bionic surface is precisely prepared by the combination method of photolithography and vacuum casting. Fig. 6(c) shows the cross-sectional view of the bionic surface. Fig. 6(d) shows the major elements of the bionic surface, which mainly consists of C, Si and O elements. The atomic percentage of C, Si and O are 44.3%, 29.7% and 26.0%, respectively.

**Fig. 7:**
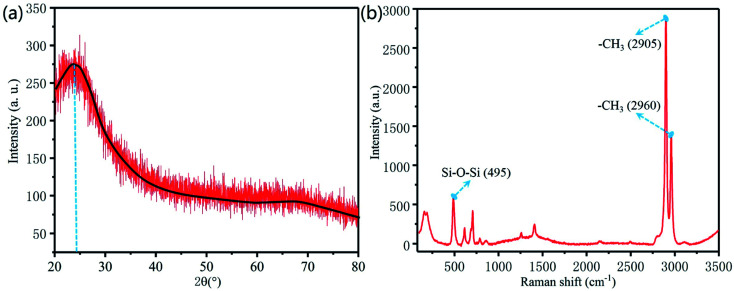
Component analysis of the bionic surface. (a) X-ray analysis (b) Raman analysis.

Fig. 7 shows the phase analysis of X-ray diffraction (XRD) and Raman spectra of the bionic surface. The X-ray diffraction of the bionic surface reaches its peak value at 24.5°, as shown in Fig. 7(a). According to Joint Committee on Powder Diffraction Standards (JCPDS), this peak value matches the PDMS diffraction peak. The peak values appear at 2905 cm^−1^ and 2960 cm^−1^, which are associated with the symmetric and anti-symmetric stretching vibration of –CH_3_, as exhibited in Fig. 7(b). The peak value at 495 cm^−1^ corresponds to the Si–O–Si symmetric stretching.^25^

### 3.2 Numerical simulation results

In the flow rate range of from 1 to 9 m s^−1^, the pressure drag, viscous drag and total drag were figured out by numerical simulation, as exhibited in Table 1. It can be concluded that the drag force gradually increases with the increase of fluid velocity. According to eqn (4),^26^ the drag reduction rate of the bionic surface can be calculated.4
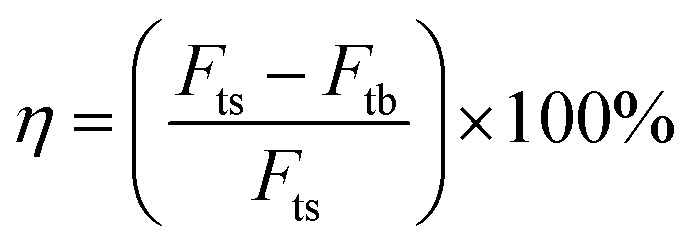
where *η* is drag reduction rate, *F*_ts_ is the resistance of the smooth surface, *F*_tb_ is the resistance of the bionic surface.

**Table 1:** Comparison between the resistance of the smooth surface and bionic surfaces

Velocity (m s^−1^)		1	3	5	7	9
Smooth surface	Pressure drag (*N*)	6.8164 × 10^−7^	4.4220 × 10^−6^	1.1045 × 10^−5^	2.0320 × 10^−5^	3.2089 × 10^−5^
	Viscous drag (*N*)	3.2640 × 10^−6^	1.5032 × 10^−5^	3.1868 × 10^−5^	5.2718 × 10^−5^	7.7002 × 10^−5^
	Total drag (*N*)	3.9456 × 10^−6^	1.9454 × 10^−5^	4.2913 × 10^−5^	7.3037 × 10^−5^	1.0909 × 10^−4^
Bionic surface	Pressure drag (*N*)	1.3833 × 10^−6^	8.2530 × 10^−6^	2.0735 × 10^−5^	3.9124 × 10^−5^	6.3666 × 10^−5^
	Viscous drag (*N*)	2.3579 × 10^−6^	9.9765 × 10^−6^	2.0001 × 10^−5^	3.1946 × 10^−5^	4.5565 × 10^−5^
	Total drag (*N*)	3.7412 × 10^−6^	1.8230 × 10^−5^	4.0737 × 10^−5^	7.1070 × 10^−5^	1.0923 × 10^−4^
Drag reduction rate (%)		5.18	6.29	5.07	2.69	−0.13

**Fig. 8:**
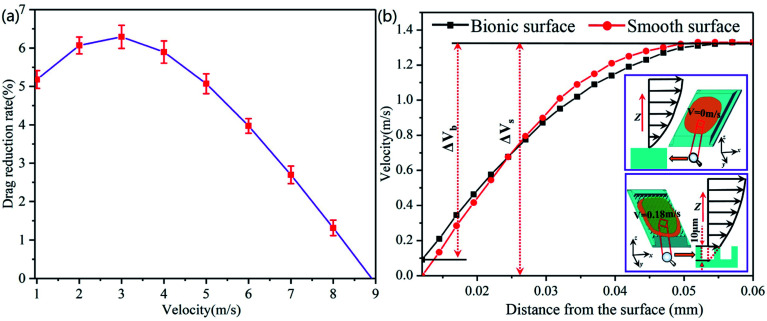
The result of numerical analysis. (a) Drag reduction rate of the bionic surface. (b) The velocity gradient of bionic surface and smooth surface.

As shown in Fig. 8(a), the drag reduction rate of the bionic surface gradually increases with the increase of velocity from 1.0 m s^−1^ to 3.0 m s^−1^, and the maximum drag reduction rate is 6.29% when velocity is 3.0 m s^−1^. However, the drag reduction rate rapidly declines with the increase of water flow velocity from the velocity of 3.0 m s^−1^ to 9.0 m s^−1^, and the bionic surface will lose the drag reduction function after 9.0 m s^−1^. Fig. 8(b) shows the near-wall flow velocity of the bionic surface and smooth surface, and the velocity gradient Δ*V*_b_ is less than Δ*V*_s_ (Δ*V*_b_ is the velocity gradient of bionic surface, and Δ*V*_s_ is the velocity gradient of the smooth surface). When the inlet water flow velocity is 1 m s^−1^, the water flow velocity of the bionic surface is 0.18 m s^−1^. The velocity slip appears on the bionic surface, and the slip length is 10 μm. On the contrary, there is no velocity slip on the smooth surface, and the water flow velocity of the smooth surface is 0 m s^−1^. Therefore, the existence of microstructure dramatically reduce the thickness of the viscous sublayer and transition layer. The velocity on the bionic surface quickly increases and finally reaches the velocity of the center zone. Hence, the shear stress of the bionic surface is lower than that of a smooth surface, according to eqn (5).^27^5
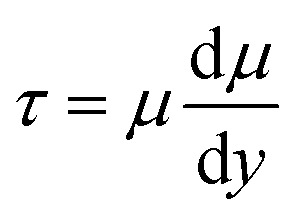
where*τ* is shear stress, *μ* is the dynamic viscosity of the fluid and 
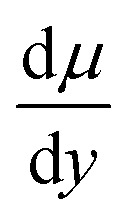
 is the velocity gradient.

**Fig. 9:**
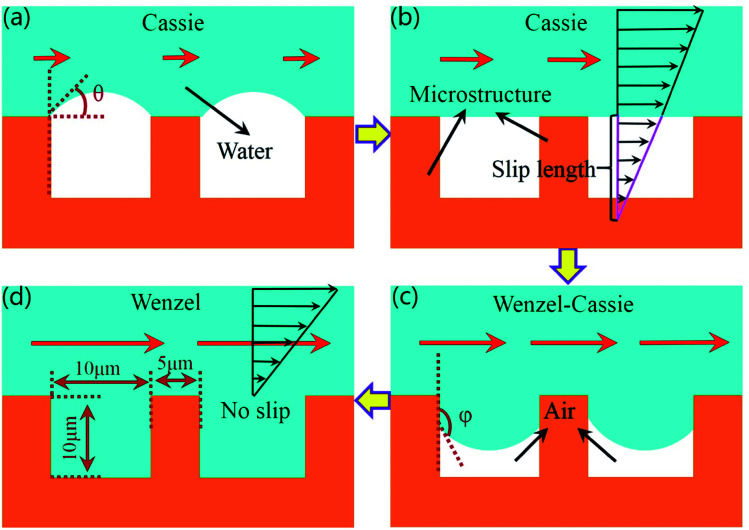
The contact state between liquid and wall. (a) Depinned-out state. (b) Pinned state. (c) Pinned-in state. (d) Fully wetted state.

When the water flow velocity on the bionic surface is lower than 3 m s^−1^, the state between the bionic surface and the water flow is Cassie state.^28^ Due to the effect of surface tension, the water flow cannot easily immerse into the gaps between papillae, and the air is trapped in the gaps, as illustrated in Fig. 9(a). The angle *θ* between air and water will gradually decrease with the increase of flow rate, and the drag reduction rate also increases. When the water pressure is equal to the surface tension, the angle between the air and the liquid is *θ* = 0, as revealed in Fig. 9(b). When the drag reduction rate reaches the maximum, the length of the velocity slip also reaches the maximum. The liquid will gradually immerse into the gaps between papillae as the flow velocity continues to increase. The contact state between the bionic surface and the fluid changes to the Wenzel–Cassie state, as shown in Fig. 9(c). As the flow velocity continues to increase, the angle between air and liquid increases, and the drag reduction rate decreases gradually. The fluid fully enters into the gaps between papillae, as shown in Fig. 9(d). The contact state between the fluid and bionic surface changes to the Wenzel state, and the velocity slip on the bionic surface will disappear. Under Wenzel state, the existence of microstructure hinders the water flowing, therefore the drag reduction effect of bionic surface disappears.

### 3.3 Experiment results

By using the water tunnel, the drag reduction effect of the bionic surface was measured. Results show that the maximum drag reduction rate of the bionic surface is 7.26% at velocity of 3.0 m s^−1^, which is a little different from the numerical simulation result. There are several reasons. First, the grid density in the numerical simulation can affect the accuracy of numerical analysis results. Second, in the actual water tunnel experiment, there are inevitably some tiny bubbles in the water flow. Even if the bubble removal treatment is carried out before the experiment, there is still difference from the pure water medium used in the simulation analysis. Last, the accuracies of valves and sensors in the experimental device also may affect the results. In summary, these factors lead to the difference between the numerical analysis and the experimental results. However, both numerical simulation and experiment results show the same trend of drag reduction.

**Fig. 10:**
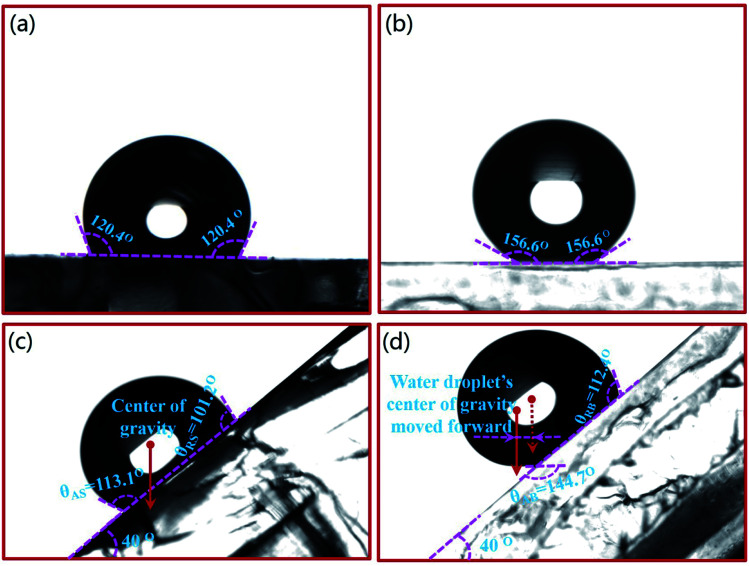
The wettability of smooth surface and bionic surface. (a) Static contact angle of smooth surface (b) Static contact angle of bionic surface (c) Rolling state of droplet on a smooth surface (d) Rolling state of droplet on the bionic surface.

In the previous studies,^29^ the result shows that the contact angle between water droplets and PDMS surface decreases gradually with the increase of temperature. When the temperature is between 0 °C and 40 °C, the contact angle is about 153°, which almost remains same. When the temperature is between 40 °C and 60 °C, the contact angle decreases gradually to 110°. In this study, the experiments were carried out at 25 °C, and the static contact angles of water droplets on bionic surface and smooth surface were measured through the contact angle instrument (DSA-25A). The static contact angle of water droplet on smooth surface is 120.4°, as shown in Fig. 10(a), and 156.6° on the bionic surface, as shown in Fig. 10(b). According to eqn (6),^30^ the surface free energy (*γ*_s_) of smooth surface and bionic surfaces are 14.26 mJ m^−2^ and 1.09 mJ m^−2^, respectively. Therefore, it can be concluded that the bionic microstructure can reduce the surface free energy and improve surface hydrophobicity.6
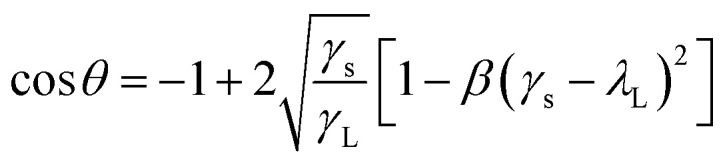
where *θ* is the contact angle, *γ*_s_ is the surface free energy of a smooth surface or bionic surface. *β* is a constant with a value of 1.1229 × 10^−4^ (m^2^ mJ^−1^)^2^ and the surface energy of water (*γ*_L_) is 72.8 mJ m^−2^.

The angle of the inclining bench is gradually increased from 0° to 25°. When the angle is 25°, the water droplet begins to roll down. The rolling angle 25° seems large for superhydrophobicity surface. The rolling angle is related to the chemical composition and the microstructure of the surface.^31^ If accurate nanostructures are achieved on the bionic surface, the rolling angle would be further reduced. Here, the water droplets rolling state was analyzed when the bench was remained at 40°. Fig. 10(c) and (d) shows the shape of the droplet rolling to point D. The shape of water droplet on a smooth surface is hemispherical, as shown in Fig. 10(c). The advancing contact angle (*θ*_AS_) and receding contact angle (*θ*_RS_) of water droplet is 113.1° and 101.2°, respectively. Conversely, the shape of the droplet rolling on the bionic surface is ellipsoidal, and the center of gravity moves forward (Fig. 10(d)). The advancing contact angle (*θ*_AB_) and receding contact angle (*θ*_RB_) of the water droplet is 144.7° and 112.4°, respectively, as shown in Fig. 10(d). It is find that the advancing contact angle and receding contact angle of the water droplet are significantly lower than the static contact angle on the bionic surface. The rolling speed of the water droplet increases and immerses into the papillae gaps gradually. The surface free energy of smooth surface is 13.17 mJ m^−2^ higher than that of bionic surface. However, the surface free energy of the bionic surface and adhesion of water droplet is lower, and the rolling speed of the water droplet is faster. The existence of microstructure reduces the contact area between the droplets and the surface. Due to the surface tension, the water droplet cannot completely immerse into the papillae gaps when it rolls from point A to point D. The contact state between water droplet and the bionic surface is Cassie or Wenzel–Cassie state, and air is trapped in the papillae gaps. Therefore, the existence of the microstructure reduces the resistance of the bionic surface.

## 4. Conclusions

In this work, the microstructure of lotus leaf surface was taken as the research objective. Trough numerical simulation and practical experiments, the wettability and drag reduction characteristics of microstructure were studied. The main conclusions are as follows:

(1) The numerical simulation showed that the drag reduction rate of the bionic surface generally increases with the increase of fluid velocity from 1.0 m s^−1^ to 3.0 m s^−1^. The drag reduction rate reaches a maximum of 6.29% at a flow velocity of 3.0 m s^−1^. With the increase of velocity, the drag reduction rate begins to decline. The bionic surface begins to lose its drag reduction effect at the flow velocity of 9.0 m s^−1^.

(2) The drag reduction mechanism of the bionic surface is explored. The velocity gradient of the bionic surface is lower than that of smooth surface. The velocity slip occurs on the bionic surface, and the slip length is 10 μm. The contact states between bionic surface and water flow change from Cassie to Wenzel–Cassie state, finally keep in the state of Wenzel with the increase of flow rate.

(3) The static contact angle of the droplet on bionic surface and smooth surface is 156.6° and 120.4°, respectively. The surface free energy of the bionic surface and smooth surface is 1.09 mJ m^−2^ and 14.26 mJ m^−2^, respectively.

(4) In the inclining bench experiment, the water droplets on the smooth surface are hemispherical, but ellipsoidal on the bionic surface, and the center of gravity of water droplet moves forward. The *θ*_AB_ and *θ*_RB_ of water droplet on the bionic surface are 144.7° and 112.4°, and the *θ*_AS_ and *θ*_RS_ of the water droplet on the smooth surface are 113.1° and 101.2°.

This work provides theoretical foundation and new ideas for the drag reduction of underwater vehicles, oil/gas pipelines, *etc.*

## Author contributions

H. W. and L. W. contributed to the writing of the manuscript, result discussion and the establishment of the model. G. L. and L. C. contributed to the experimental data processing. Y. S. and C. L. contributed to discuss the whole scheme and experimental analysis.

## Conflicts of interest

There are no conflicts to declare.

## Supplementary Material
